# Frequency of Chromosomally-Integrated Human Herpesvirus 6 in Children with Acute Lymphoblastic Leukemia

**DOI:** 10.1371/journal.pone.0084322

**Published:** 2013-12-26

**Authors:** Annie Gravel, Daniel Sinnett, Louis Flamand

**Affiliations:** 1 Division of Infectious Diseases and Immunity, CHU de Quebec Research Center, Quebec, Canada; 2 Centre de recherche, Service d'hématologie-oncologie, CHU Sainte-Justine, Département de pédiatrie, Université de Montréal, Montreal, Quebec, Canada; 3 Department of Microbiology, Infectious Diseases and Immunology, Laval University, Quebec, Quebec, Canada; Queen's University, Canada

## Abstract

**Introduction:**

Human herpesvirus 6 (HHV-6) is a ubiquitous pathogen infecting nearly 100% of the human population. Of these individuals, between 0.2% and 1% of them carry chromosomally-integrated HHV-6 (ciHHV-6). The biological consequences of chromosomal integration by HHV-6 remain unknown.

**Objective:**

To determine and compare the frequency of ciHHV-6 in children with acute lymphoblastic leukemia to healthy blood donors.

**Methodology:**

A total of 293 DNA samples from children with pre-B (n = 255), pre-pre-B (n = 4), pre-T (n = 26) and undetermined (n = 8) leukemia were analyzed for ciHHV-6 by quantitative TaqMan PCR (QPCR) using HHV-6 specific primers and probe. As control, DNA samples from 288 healthy individuals were used. Primers and probe specific to the cellular GAPDH gene were used to estimate integrity and DNA content.

**Results:**

Out of 293 DNA samples from the leukemic cohort, 287 contained amplifiable DNA. Of these, only 1 (0.35%) contained ciHHV-6. Variant typing indicates that the ci-HHV-6 corresponds to variant A. None of the 288 DNA samples from healthy individuals contained ciHHV-6.

**Conclusion:**

The frequency of ciHHV-6 in children with acute lymphoblastic leukemia is similar (p = 0.5) to that of healthy individuals. These results suggest that acute lymphoblastic leukemia does not originate as a consequence to integration of HHV-6 within the chromosomes.

## Introduction

Human herpesvirus-6A (HHV-6A) and HHV-6B are two closely related but distinct viruses belonging to beta-herpesvirus subfamily [1], [2]. HHV-6B is a highly prevalent virus and the etiologic agent of roseola infantum, also known as the sixth rash-causing childhood disease [3]. HHV-6B is also a source of concern in hematopoietic transplant recipients where viral reactivations are linked with a variety of medical conditions ranging from mild to life threatening. While HHV-6B is present in nearly 100% of the world's population, HHV-6A appears to be less frequent in Japan, North America, and Europe. Interestingly, HHV-6A is the predominant variant associated with viremic infant-infections in sub-Saharan Africa [4].

HHV-6 infection generally follows the classical herpesvirus replicative cycle with the release of infectious virions and destruction of the infected cells. For reasons that are unclear, HHV-6 can also integrate the host DNA leading to an “unclassic” form of latency. Work by Arbuckle et al teaches us that *in vitro* infection can lead to HHV-6 chromosomal integration (ciHHV-6) with a possibility to reactivate and produce infectious HHV-6 [5]. The first *in vivo* reports of ciHHV-6 date to the early to mid-1990s, when Luppi et al. detected the presence of a partial and possibly full-length integrated HHV-6 genome in the DNA of freshly isolated peripheral blood mononuclear cells (PBMC) [6]–[8]. In subjects with ciHHV-6, the integrated virus is present at 1 copy/cell suggesting hereditary transmission [9]. Reports estimate that HHV-6 is integrated in the telomeres of approximately 0.2–1% of individuals from Europe, USA and Japan [6], [9]–[13]. By extrapolation, this means that nearly 70 million individuals carry a 170 kilobase insertion (HHV-6 genome size) within their telomeric region. Data so far indicate that HHV-6 genome integration can occur in different chromosomes but invariably takes place in the sub-telomeric or telomeric regions. It is now well established that the self-renewal potential of cells is directly proportional to telomere lengths and telomerase activity [14], [15]. The loss of telomere function can cause cell cycle arrest and apoptosis. Inversely, the loss of telomere function can also lead to genetic instability and cancer progression. It is also known that loss of telomere functions preferentially occurs on the shortest telomeres[16]. When the number of telomeric repeated sequence (TRS) falls below 13, chromosomal instability is observed [17]. Several diseases are linked with telomere dysfunctions and/or telomerase mutations such as hematopoietic dysfunction, pulmonary fibrosis, liver disease, degenerative diseases and cancer [18]–[29]. Alterations within telomeric regions are therefore a likely cause for cellular dysfunctions linked to diseases but many of the factors affecting telomeres integrity remain to be identified. The HHV-6 integration mechanisms and the biological/medical consequences resulting from this telomeric alteration remain largely unknown but interestingly, ciHHV-6 is 2.3× more frequent (p<0.001) in diseased (various diseases) individuals relative to healthy ones [30]. Interestingly, integration of Marek's disease virus (an alpha-herpesvirus of chicken) into the telomeres is linked with the development of T cell lymphoma [31], [32]. In this study, we wanted to determine and compare the frequency of ciHHV-6 in children with acute lymphoblastic leukemias (ALL) to healthy blood donors in order to determine whether ciHHV-6 represents a risk factor for such blood malignancies.

## Materials and Methods

### DNA Samples

Our cohort consisted of 293 childhood ALL patients and 288 healthy controls. Study subjects were all French-Canadians of European descent from the established Quebec Childhood ALL (QcALL) cohort [33]. Incident cases were diagnosed in the Division of Hematology-Oncology at the Sainte-Justine University Health Center (SJUHC), Montreal, Canada, between October 1985 and November 2006. Healthy controls consisted of a group of French-Canadian newborns and adults recruited at the SJUHC. The CHU Sainte-Justine Institutional Review Board approved the research protocol, and written informed consent was obtained from all participating individuals and/or their parents. Tables 1 and 2 list the characteristics of each group.

**Table 1:** Characteristics of control subjects analyzed for ciHHV-6.

Healthy subject Characteristics	Number (%)
Total number of subjects	288
	
Gender	
Male	147 (51.0)
Female	141 (49.0)
	
Age (years)	
Mean	25.2
Median	28.7
Standard deviation	5.8 (0 to 69.75)

**Table 2:** Characteristics of leukemic subjects analyzed for ciHHV-6.

Leukemic Patient Characteristics	Number (%)
Total number of subjects	293
	
Gender	
Male	166 (56,7)
Female	127 (43,3)
	
Age (years)	
Mean	6,0
Median	4,5
Standard deviation	1.5 (0.4 to 17.9)
	
Immunophenotype	
Pre-B	255 (87.0)
Pre-pre B	4 (1.4)
Pre-T	26 (8.9)
N/D	8 (2.7)
	
Age group (years)	
≤ 1	5 (1.7)
1-10	230 (78.5)
>10	52 (17.7)
N/D	6 (2.1)
	
Hyperdiploidy	
Positive	98 (33.4)
Negative	173 (59.0)
N/D	22 (7.6)
	
Chromosomal translocations	
Absence of translocation	140 (47.8)
t(12;21)	40 (13.7)
Other	27 (9.2)
N/D	86 (29.3)
	
Normal ploidy and absence of	79 (27.0)
chromosomal translocations	

### Real-Time Quantitative PCR (QPCR)

QPCR analyses were performed on a Rotor-Gene Q (Qiagen) with the Rotor-Gene Multiplex PCR Kit (Qiagen) for GAPDH and U65-U66 detection [34]. The following primer pairs have been used: GAPDH gene forward primer, 5′-CGAGATCCCTCCAAAATCAA-3′; GAPDH gene reverse primer, 5′-TTCACACCCATGACGAACAT-3′; GAPDH gene probe, 5′-hexachloro-6-carboxyfluorescein-TGGAGAAGGCTGGGGCTCAT-black-hole-quencher-1-3′; U65-U66 gene forward primer, 5′-GACAATCACATGCCTGGATAATG-3′; U65-U66 gene reverse primer for HHV-6A/B, 5′-TGTAAGCGTGTGGTAATGGACTAA-3′ ; U65-U66 gene reverse primer specific for HHV-6A, 5′-TGGTAATGGACTAATTGTGTGTTGTTTTA-3′; U65-U66 gene reverse primer specific for HHV-6B, 5′-TGGTAATGGACTAAGTGTGCGTTATTTTC-3 ; U65-U66 gene probe, 5′-6-carboxyfluorescein-AGCAGCTGGCGAAAAGTGCTGTGC-black-hole-quencher-1-3′.

Statistical analysis. To frequency of ciHHV-6 in healthy and leukemic patients was compared using the Fisher's exact test.

## Results and Discussion

Detection and identification of individuals carrying ciHHV-6 is relatively simple. Since these individuals carry at least one copy of HHV-6 genome per cell, there is more than a thousand-fold difference in the number of HHV-6 DNA copies/μg of DNA between ciHHV-6 individuals and those that harbor latent episomal (non-integrated) HHV-6 [35]. In fact, the mean number of HHV-6 copies/μg of cellular DNA in individuals with ciHHV-6 is approximately 10^6^ while those with post-natal HHV-6 acquisition are in the range of 10^3^ copies/μg of cellular DNA [10], [35], [36]. Using leukocytes or any other source of cellular DNA and quantitative polymerase chain reaction (QPCR) assay, it is therefore very easy to identify and distinguish ciHHV-6 from non ciHHV-6. We use a published and validated TaqMan-based procedure [37] to detect HHV-6A and HHV-6B This assay can be easily modified to discriminate HHV-6A from HHV-6B [38]. Lastly, as part of a multicenter study conducted across the world, we have reported that this PCR assay is a sensitive and reliable method to detect and quantitate HHV-6 [34].

Using these tools, we have screened 581 DNA samples from children with ALL and healthy donors of French-Canadian origin by QPCR using specific primers for HHV-6 U65-U66 gene and GAPDH, as a housekeeping gene. GAPDH amplification was used to assess the quality of the DNA samples and used for normalization. The demographics and characteristics of the control and leukemic subjects are presented in tables 1 and 2, respectively. The age difference between the leukemic subjects (median 4.5 years) and the control subjects (28.1 years) is not an issue considering that ciHHV-6 is inherited [9]. As presented in table 3, all healthy subjects were negative (<10 copies/50 ng of DNA) for the presence of HHV-6 DNA. In our assay, which has a limit of detection of 10 HHV-6 DNA copies, we used 50 ng of genomic DNA meaning that we could only detect subjects with ≥200 copies of HHV-6/μg of DNA. The fact that all healthy subjects were negative for HHV-6 DNA in therefore not unexpected considering thelow incidence (10-15%) and low viral loads (median of 62 HHV-6 copies/μg of genomic DNA) reported [39]

**Table 3:** QPCR results.

Healthy Subjects	Number (%)
Total number of subjects	288
	
QPCR	
HHV-6 positive	0 (0)
HHV-6 negative	288 (100)
	
GAPDH positive	288 (100)
GAPDH negative	0 (0)
	
Leukemic Patients	
Total number of subjects	293
	
QPCR	
HHV-6 positive	11 (3.8)
HHV-6 negative	276 (96.2)
	
GAPDH positive	287 (97.9)
GAPDH negative	6 (2.1)*

* All GAPDH-negatives were also HHV-6-negative.

In contrast to healthy individuals, DNA samples taken at time of diagnostic from 11 leukemic patients (10 pre-B and 1 pre-T) were positive for HHV-6 DNA (table 3). We determined the HHV-6 copy number in the leukemic patients using a standard curve generated with a plasmid carrying a portion of the U65-U66 gene [37]. We also analyzed all patients using our standard curve for GAPDH, made with a plasmid carrying GAPDH gene. All the data were normalized using the GAPDH copy number. The HHV-6 copy number/μg in ten of these patients varied between 90 and 2410 copies/μg of DNA (median = 320) (table 4). We analyzed the HHV-6 positive patients with primer pairs that discriminate between HHV-6A and HHV-6B. Eight carried HHV-6B and 1 HHV-6A (one could not be not determined). The detection of low HHV-6 viral loads in a small proportion of ALL patients has been observed previously [40], [41]. Of these 11 samples, one (P451) had a HHV-6 copy number consistent with ciHHV-6. Relative to a gene such as GAPDH that is present in two copies in a diploid genome, ciHHV-6 is present at one copy per cell, unless both parents carry ciHHV-6, a rather rare occurrence [9]. As presented under table 4, P451 had 511 650 HHV-6A DNA copies per μg of DNA. As positive control, we used DNA from a subject with confirmed ciHHV-6 [5].

**Table 4:** Detailed analyses of HHV-6 DNA positive patients.

Leukemic Patients	HHV-6 copies*	HHV-6	% blasts	Diagnostic	Diagnosis age (yr)
P101	2000	B	ND	Pre-B	8,3
P331	680	B	97,5	Pre-B	4,6
P451	511650	A	82,0	Pre-B	6,8
P454	360	ND	83,5	Pre-B	6,2
P458	2410	B	99,0	Pre-B	3,4
P459	770	A	97,5	Pre-B	2,5
P462	90	B	43,2	Pre-B	8,7
P467	150	B	96,5	Pre-B	2,8
P468	280	B	100	Pre-B	2,8
P478	150	B	90,5	Pre-T	16,9
P484	150	B	89,0	Pre-B	11,0
Positive controlciHHV-6	656180	A	NA	NA	NA

* normalized with GAPDH housekeeping gene (/μg of DNA).

ND: Not determined; NA: not applicable.

The frequency of ciHHV-6 varies between 0.2-3% depending of the geographic area, the sample sized analyzed and disease conditions (reviewed in [30]). However, when only considering studies with sampling size above 500 subjects, the incidence of ciHHV-6 in healthy individuals from the US or the UK is approximately 1%. HHV-6B represents the integrated virus in two thirds of ciHHV-6 cases [42]. In our cohort, out of 575 individuals tested, 1 ciHHV-6+ individual (0,17%) was detected. The frequency of ciHHV-6 in the Province of Quebec (Canada) therefore appears lower that that observed in US and UK. Furthermore, the one ciHHV-6+ sample detected corresponds to HHV-6A. How can these differences be explained? Our sampling size is certainly one limiting factor preventing us from ascertain with confidence whether the incidence of ciHHV-6 is truly different for the US of the UK. Statistical analysis indicates that the incidence of ciHHV-6 in Quebec does not differ (p = 0.13) from that reported by Hall et al [43]. By expanding the number of subjects, a more precise estimate on the incidence of ciHHV-6 within the population of Quebec would be obtained.

Of interest, the French-Canadian population is considered genetically more homogeneous than other population of European descent due to a limited number of settlers (e.g. founder effect) combined with a large demographic expansion[44]. At present, the Quebec population comprises 7.8 million residents, of which ∼80% are French Canadians. The apparent lower frequency of ciHHV-6+ individuals in the province of Quebec could be consequent to initial lower incidence of ciHHV-6 within the settlers.

## Conclusion

The frequency of ciHHV-6 in children with acute lymphoblastic leukemia is similar (p = 0.5) to that of healthy individuals. Our results are in accordance with those of Hubacek et al that reported on the frequency of ciHHV-6 in children with acute lymphoblastic or myeloid leukemia from the Czech republic [40]. These results suggest that childhood ALL does not originate as a consequence to integration of HHV-6 within the chromosomes.
